# Advances in Carbon-Based Microwave Absorbing Materials

**DOI:** 10.3390/ma15041359

**Published:** 2022-02-12

**Authors:** Yunchen Du

**Affiliations:** MIIT Key Laboratory of Critical Materials Technology for New Energy Conversion and Storage, School of Chemistry and Chemical Engineering, Harbin Institute of Technology, Harbin 150001, China; yunchendu@hit.edu.cn

Electromagnetic (EM) pollution has been evolving as one of the most concerning environmental problems in current society, due to the extensive application of EM technology, from household electronic apparatuses to wireless base stations, as well as military radars. Although both shielding and absorption are effective for resistance toward EM interference, the latter has received much more attention in recent years because it can convert EM energy and dissipate surplus EM waves [1]. Microwave absorbing materials (MAMs) are usually defined as a kind of functional material that can interact with the magnetic or electronic branch of incident EM waves and, thus, weaken EM energy greatly. Magnetic metals and ferrites are early utilized as MAMs, while high density and poor environmental tolerance restrain their practical applications to some extent, especially under the context of increasing requirements for the oncoming generation of MAMs. Among various candidates, carbon materials show great potential, in virtue of their good chemical stability, tailorable dielectric property, diversified microstructure and morphology, and broad compatibility with other EM components [2]. Rational design on carbon-based MAMs is becoming a hot topic in the field of EM absorption.

Conventional carbon black and graphite are rarely involved in carbon-based MAMs, because they easily suffer from impedance mismatching, and their relatively large size may induce serious phase separation in final composites. In contrast, carbon nanotubes (CNTs) and carbon nanofibers (CNFs) are more popular substrates for carbon-based MAMs, and their unique one-dimensional configuration has been verified to be favorable for accelerating electron transfer and consolidating conductivity loss [3]. It is common to produce dielectric–magnetic synergy by decorating CNTs/CNFs with magnetic nanoparticles, while the synergy from two dielectric components is also concerned in recent advances. For example, Li et al. coated CNTs with SiC, and the resultant composite (P-600) displayed much better oxidation resistance and stable dielectric properties than pristine CNTs [4]. After heat treatment at 400 °C for 200 h, the mass loss of P-600 was less than 1.86%, and the complex permittivity was almost unchanged. This meaningful study even opens up the possibility for the development of carbon-based MAMs applied at high temperature. In addition to the decoration or coating of CNTs/CNFs, there has been an alternative strategy to construct carbon-based MAMs, that is, the in-situ growth of CNTs on a specific substrate. Liu et al. employed hierarchical Ni@C microspheres as the substrate to induce CNTs growth, and the emergence of CNTs enhanced the attenuation ability of the composites, and strong reflection loss (−41.5 dB) and wide qualified absorption bandwidth (5.2 GHz) could be achieved, with the absorber thickness less than 2.0 mm [5].

The advent of graphene boosts the research of carbon-based MAMs significantly, and there have been over 4000 published papers on the fabrication of graphene-based MAMs, since its first report in 2011 [6]. The typical two-dimensional morphology of graphene not only facilitates the deposition of various nanoparticles, but also offers considerable possibilities to strengthen interfacial polarization, by creating face-to-face heterojunctions with other two-dimensional guests. Qing et al. assembled N-doped graphene and Ti_3_C_2_ nanosheets, and they found that the composite had much better microwave absorption performance than individual N-doped graphene or Ti_3_C_2_ nanosheets, whose minimum reflection loss and qualified absorption bandwidth reached up to −52.0 dB and 7.1 GHz, with an absorber thickness of 1.4 mm [7]. Recent progress indicates that there is great interest in the construction of carbon-based MAMs, with three-dimensional graphene aerogel as the main scaffold, because such an architecture suppresses the re-stacking of graphene nanosheets, and more importantly, the extremely high porosity creates longer pathways of incident EM waves through multiple reflections and scatterings, and interfacial defects and functional groups, together with three-dimensional conductive networks, also make solid contribution to EM energy conversion [8]. It is of note, that most studies still focus on the surface modification of three-dimensional graphene aerogel, while the spatial embedment of EM particles is still inaccessible.

Apart from these common members in the carbon family, carbon microspheres have also earned a place in the field of EM absorption. On one hand, their uniform spherical morphology makes it easy to disperse them into many solvents or resin matrix, and the serious agglomeration or re-stacking in CNTs/CNFs and graphene will not occur; on the other hand, the amorphous nature of carbon microspheres can create good impedance matching and bring more space for regulation of EM properties [9]. There are two popular strategies to reinforce EM absorption performance of carbon microspheres, i.e., hollow engineering and magnetic particle embedding. Hollow cavities have similar functions to three-dimensional graphene aerogel, wherein multiple reflections and scatterings promote EM energy conversion. It was found that multi-chamber carbon microspheres had better impedance matching than hollow carbon microspheres, and stronger attenuation ability than solid carbon microspheres [10]. The advantages of magnetic particles embedding are not limited in the formation of magnetic loss, and actually, carbon shells may also suppress the agglomeration of magnetic nanoparticles and subsequent skin effect, thus, leading to better magnetic–dielectric synergy [11].

Since the shape-preserving transformation of metal-organic frameworks (MOFs), it has rapidly developed into a dominant method for carbon-based MAMs. MOFs-derived, carbon-based materials usually possess some favorable features for EM absorption, including uniform morphology, porous microstructure, and well-dispersed metal nanoparticles. Although some significant achievements have been made in MOFs-derived carbon-based MAMs, a dilemma still hinders their popularization; that is, their composition and microstructure are in over-reliance on the corresponding MOFs precursors, and not in the optimum state for EM absorption. At present, composition optimization and microstructure design of MOFs-derived carbon-based MAMs are becoming a hotspot in the related field [12]. Some additional carbon materials and magnetic particles are widely employed to modulate EM properties of MOFs-derived carbon-based MAMs. Wang et al. conducted the growth of Fe-doped ZIF-67 on the surface of porous cocoon-like rGO aerogel and benefited from the improvements on both chemical composition and microstructure, the resultant Fe-Co/NC/rGO exhibited qualified absorption bandwidth as broad as 9.3 GHz [13].

Biomass is considered as a kind of promising carbon source to construct carbon-based MAMs, not only for its abundant reserves and low cost, but more importantly for the fact that it ensures the formation of hierarchically porous microstructure in final carbon materials. In an effort to achieve better EM absorption performance, EM properties of biomass-derived carbon materials are generally regulated by introducing additional dielectric and magnetic components, through pre-impregnation or post-treatment methods [14]. To date, there is no obvious gap in EM absorption between biomass-derived carbon-based materials and other counterparts, which again validates the bright prospect of biomass in this field. Although carbon-based MAMs have made great progress in recent years, some challenges related to their practical application still remain unresolved, and the most concerning of these will be highly efficient, low-frequency absorption and ultrabroad response bandwidth within a small absorber thickness in the future.
